# Immunogenicity of Adjuvanted Psoralen-Inactivated SARS-CoV-2 Vaccines and SARS-CoV-2 Spike Protein DNA Vaccines in BALB/c Mice

**DOI:** 10.3390/pathogens10050626

**Published:** 2021-05-19

**Authors:** Appavu K. Sundaram, Daniel Ewing, Zhaodong Liang, Vihasi Jani, Ying Cheng, Peifang Sun, Kanakatte Raviprakash, Shuenn-Jue Wu, Nikolai Petrovsky, Gabriel Defang, Maya Williams, Kevin R. Porter

**Affiliations:** 1 Viral and Rickettsial Diseases Department, Naval Medical Research Center, Silver Spring, MD 20910, USA; daniel.f.ewing.civ@mail.mil (D.E.); zhaodong.liang.ctr@mail.mil (Z.L.); vihasi.s.jani.ctr@mail.mil (V.J.); ying.cheng4.ctr@mail.mil (Y.C.); peifang.sun2.civ@mail.mil (P.S.); kanakatte.s.raviprakash.civ@mail.mil (K.R.); shuenn-jue.l.wu.civ@mail.mil (S.-J.W.); 2 Henry M. Jackson Foundation for the Advancement of Military Medicine, Bethesda, MD 20817, USA; 3 Leidos, 1750 Presidents St, Reston, VA 20190, USA; 4 Vaxine Pty Ltd., Warradale, SA 5042, Australia; nikolai.petrovsky@flinders.edu.au; 5 Naval Research Laboratory, Washington, DC 20375, USA; maya.williams@nrl.navy.mil; 6 Infectious Diseases Directorate, Naval Medical Research Center, Silver Spring, MD 20910, USA; kevin.r.porter3.civ@mail.mil

**Keywords:** SARS-CoV-2, COVID-19, psoralen-inactivated SARS-CoV-2 vaccine, anti-SARS-CoV-2-neutralizing antibodies, conformational epitopes of surface antigens, 4′-aminomethyl-4,5′,8-trimethylpsoralen (AMT), Advax-2, DNA vaccine, prime-boost

## Abstract

The development of a safe and effective vaccine to protect against COVID-19 is a global priority due to the current high SARS-CoV-2 infection rate. Currently, there are over 160 SARS-CoV-2 vaccine candidates at the clinical or pre-clinical stages of development. Of these, there are only three whole-virus vaccine candidates produced using β-propiolactone or formalin inactivation. Here, we prepared a whole-virus SARS-CoV-2 vaccine (SARS-CoV-2 PsIV) using a novel psoralen inactivation method and evaluated its immunogenicity in mice using two different adjuvants, alum and Advax-2. We compared the immunogenicity of SARS-CoV-2 PsIV against SARS-CoV-2 DNA vaccines expressing either full-length or truncated spike proteins. We also compared the psoralen-inactivated vaccine against a DNA prime, psoralen-inactivated vaccine boost regimen. After two doses, the psoralen-inactivated vaccine, when administered with alum or Advax-2 adjuvants, generated a dose-dependent neutralizing antibody responses in mice. Overall, the pattern of cytokine ELISPOT responses to antigen-stimulation observed in this study indicates that SARS-CoV-2 PsIV with the alum adjuvant promotes a Th2-type response, while SARS-CoV-2 PsIV with the Advax-2 adjuvant promotes a Th1-type response.

## 1. Introduction

In the past 20 years, three coronaviruses have crossed species barriers to infect humans and cause acute severe respiratory illness: severe acute respiratory syndrome coronavirus (SARS-CoV) in 2003, Middle East respiratory syndrome coronavirus (MERS-CoV) in 2012, and SARS-CoV-2 in 2019 [1,2]. Currently, there have been more than 116 million cases of SARS-CoV-2 infections and more than 2.5 million deaths worldwide due to this pandemic [3]. SARS-CoV-2 infections in humans emerged in December 2019 and have since spread throughout the world, causing COVID-19. Though the majority of SARS-CoV-2-infected patients exhibit a mild disease with recovery typically within two-to-three weeks, a significant number of patients manifest the severe form of the illness with increased mortality.

The development of a safe and effective vaccine to protect against COVID-19 is a global priority due to the current high rate of disease transmission and the high number of hospitalizations and deaths that threaten to overwhelm health systems in many countries. Most of the vaccines currently in pre-clinical and clinical trials have been designed to elicit antibodies against the SARS-CoV-2 spike protein and include advanced vaccine platforms such as messenger RNA-based vaccines, DNA vaccines, viral vector vaccines, recombinant protein vaccines, and virus-like particle vaccines [4,5]. Recently, mRNA vaccines against COVID-19, developed by BioNTech/Pfizer and Moderna, have been granted an emergency use authorization in both the UK and USA. While these vaccines show short-term protective efficacy against COVID19, it is still unclear whether targeting the spike protein alone will be sufficient to provide long-term protection against the disease [6]. Accordingly, developing a whole-virus-inactivated vaccine to target several other viral antigens including the spike protein, nucleoprotein, envelope protein, and membrane protein to elicit a broader immune response is a logical approach given that a natural infection generates immune responses to these other proteins. 

Recently, two groups have developed whole-virus-inactivated SARS-CoV-2 vaccines using β-propiolactone, and evaluated their efficacy and immunogenicity in nonhuman primates [7,8]. β-propiolactone or formalin, commonly used compounds to inactivate viruses, are potentially carcinogenic compounds that inactivate viruses at the protein and nucleic acid levels [9,10]. 

We previously investigated the use of psoralen to inactivate all four serotypes of dengue virus and evaluated the immunogenicity of psoralen-inactivated dengue virus vaccines in mice and nonhuman primates [11,12]. Psoralen is a furanocoumarin that intercalates with nucleic acids and, upon exposure to long wave ultraviolet radiation, leads to interstrand cross-links by covalently binding to pyrimidine bases [13,14,15]. Thus, psoralen inactivation serves to inactivate the virus at the nucleic acid level while presumably preserving immunogenic protein epitopes on the virus surface. 

In this article, we describe the inactivation of SARS-CoV-2 using the psoralen derivative 4′-aminomethyl-4,5′,8-trimethylpsoralen (AMT) and irradiation with long wave UV light (365 nm) to produce a purified inactivated SARS-CoV-2 vaccine (SARS-CoV-2 PsIV). The main objective of this initial mouse study was to show that SARS-CoV-2 PsIV is able to elicit antibody and T-cell responses in a mammalian model. We then compared the immunogenicity of this vaccine to SARS-CoV-2 DNA vaccines in mice. The SARS-CoV-2 PsIV vaccine was formulated with either alum or Advax-2 (Advax-CpG55.2) adjuvants. The SARS-CoV-2 DNA vaccine either encoded the full-length SARS-CoV-2 spike protein or a truncated spike protein (lacking the transmembrane region). The DNA vaccines were also used in a heterologous prime-boost approach, wherein the animals were primed with two doses of the DNA vaccine and then boosted with the SARS-CoV-2 PsIV formulated with Advax-2. Here, we report the results of these immunogenicity studies in mice.

## 2. Materials and Methods

### 2.1. Chemicals and Reagents

AMT was purchased from Cayman Chemical (Ann Arbor, MI, USA). Recombinant human serum albumin was purchased from eEnzymes, LLC (Gaithersburg, MD, USA). The Alhydrogel adjuvant (2%) was obtained from InvivoGen (San Diego, CA, USA), and the Pluronic F-127 co-polymer was obtained from Sigma Aldrich. The Advax-2 adjuvant was provided by Vaxine Pty Ltd., Adelaide, Australia. All other chemicals and reagents were purchased from Thermo Fisher Scientific (Waltham, MA, USA). Rabbit anti-SARS-CoV-2 Ab (NMRC) was used as the primary antibody for ELISA. A goat anti-rabbit IgG HRP conjugate (Pierce) was used as the secondary antibody for ELISA. ABTS One Component (Seracare Inc., Milford, MA, USA) was used as the substrate.

### 2.2. Preparation and Inactivation of SARS-CoV-2

Briefly, the SARS-CoV-2 strain nCoV/USA-WA1/2020 was propagated in Vero E6 cell cultures and harvested by centrifugation at 3000× *g* for 15 min. Then, 500 mL of the culture supernatant containing SARS-CoV-2 was treated with benzonase (an enzyme degrading free nucleic acids) to remove host cell nucleic acids in the culture supernatant, and the volume was reduced to 50 mL (concentrating) using 100 K MWCO membrane filter cassettes. The concentrated SARS-CoV-2 virus preparation was mixed with AMT at 50 µg of AMT/mL, and the resulting mixture was then treated with long wavelength UV light (λ = 365 nm) for 5 min (total energy applied = 1,445,400 µjoules). The complete inactivation of psoralen/UVA-treated SARS-CoV-2 virus was confirmed by its inability to grow in permissive cells (Vero E6 cells) by a two-passage virus amplification test. Briefly, 50 µL aliquots of the inactivated virus were used to infect cultured cells in duplicate. After incubation at 37 °C for 5–8 days, cells and culture supernatants were examined for the presence of SARS-CoV-2 antigens by an indirect immunofluorescence assay and Western blot analysis, respectively. The supernatant from this culture was then incubated with fresh Vero E6 cells for a second round of amplification and testing. Negative results (indicating the absence of virus-specific antigens) confirmed the complete inactivation of SARS-CoV-2.

### 2.3. Purification and Characterization of SARS-CoV-2 PsIV

Psoralen-inactivated SARS-CoV-2 was purified by glycerol-potassium tartrate gradient centrifugation. A stabilizer was then added to the pure SARS-CoV-2 PsIV, filtered through a 0.22 micron filter, and stored at −80 °C. The stabilizer was comprised of a final concentration of 0.5% recombinant human serum albumin, 2% Pluronic F-127, and 15% trehalose. The presence of SARS-CoV-2 antigens in the purified, inactivated virus preparation was confirmed by Western blot using SARS-CoV-2-specific anti-spike protein, anti-nucleoprotein, and anti-envelope protein antibodies. The resulting product was the purified psoralen-inactivated SARS-CoV-2 vaccine (SARS-CoV-2 PsIV). The purity of SARS-CoV-2 PsIV was assessed by gel electrophoresis followed by silver staining. SARS-CoV-2 PsIV titer (particles/mL) was determined using Virocyt 2.0.

### 2.4. DNA Vaccines

DNA sequences encoding a full-length SARS-CoV-2 (Washington strain) spike protein and a truncated spike protein (devoid of the transmembrane and cytoplasmic domain S) were separately cloned into plasmid vector VR1012 (Vical Inc., San Diego, CA, USA) [16,17]. Purified endotoxin-free (<10 U/mg of DNA) recombinant DNA constructs were used in this study.

### 2.5. Immunogenicity Assessment of SARS-CoV-2 Vaccine Candidates in Mice

The experiments reported herein were conducted in compliance with the Animal Welfare Act and in accordance with the principles set forth in the Guide for the Care and Use of Laboratory Animals, National Research Council, National Academy Press, 2011. The study protocol was reviewed and approved by the WRAIR/NMRC Institutional Animal Care and Use Committee (IACUC) in compliance with all applicable federal regulations governing the protection of animals and research. BALB/C mice (Female; 6–8 weeks old) were purchased from Charles River. During the study, mice were housed 4 per cage and fed a standard diet of commercially produced mouse chow. Groups of 4 mice were immunized with different vaccines/adjuvants by the intradermal (tail) inoculation of 50 µL doses of vaccines. SARS-CoV-2 PsIV and SARS-CoV-2 DNA vaccines were evaluated as illustrated in Table 1. Animals in group 1 served as controls and received alum on days 1, 29, and 57. Animals in group 2 were also controls and received Advax-2 alone on days 1, 29, and 57. Low and high doses of the SARS-CoV-2 PsIV vaccine, formulated with either alum or Advax-2 (as indicated in the table), were administered on days 1, 29, and 57 to groups 3, 4, 5, and 6. Animals in groups 7 and 8 received 50 µg doses of the respective DNA vaccines (DNA alone) on days 1, 29, and 57. Animals in the prime/boost groups (9 and 10) received 50 µg doses of the respective DNA vaccines (DNA alone) on days 1 and 29, and they received 10^7^ particles of SARS-CoV-2 PsIV formulated with Advax-2 on day 57. Blood was drawn from all animals on days 0, 29, and 56. On day 71, blood was collected from all the animals before euthanasia to harvest spleens to measure T-cell responses. Day 56 and 71 serum samples from each animal were tested for the presence of SARS-CoV-2-neutralizing antibodies by microneutralization assay.

### 2.6. SARS-CoV-2 Microneutralization Assay

Anti-SARS-CoV-2-neutralizing antibodies in serum were assayed using a microneutralization test. Two hundred TCID_50_ of SARS-CoV-2 were incubated with two-fold dilutions of serum samples in 96-well plates for 1 h at 37 °C. Vero81 cells (2 × 10^4^) were then added to each well and incubated at 37 °C for 84 h. After 84 h, the cells were fixed, and SARS-CoV-2 was measured by quantitating the spike protein using SARS-CoV-2-specific anti-spike protein antibody in a standard ELISA format. The highest serum dilution that resulted in an ≥80% reduction in absorbance when compared to control was determined as an 80% microneutralization titer (MN_80_).

### 2.7. T-Cell Assays

#### 2.7.1. Extraction of Splenocytes from Mouse Spleen

Mouse spleens were harvested and placed in 6-well tissue culture plates containing RPMI-1640 media with 10% fetal bovine serum, a 1% penicillin–streptomycin mixture, and 50 µM 2-mercaptoethanol. A small incision was made in the splenic capsule to allow for the diffusion of splenocytes into the media, which was facilitated by the application of gentle pressure using a 1 mL syringe. The media were gently mixed to break up any cell aggregates and transferred to a 50 mL centrifuge tube through a 70 µm cell strainer. The centrifuge tubes were spun at 1600 rpm for 8 min at 4 °C, and the resulting pellet was suspended in 40 mL of chilled phosphate-buffered saline (PBS). The tubes were centrifuged again at 1280 rpm for 8 min at 4 °C, and the pellet was resuspended in PBS before being counted for recovery and viability. Fresh spleen cells were used for IFN-γ and IL-2 ELISPOT assays, and the remaining cells were frozen at a concentration of 1 × 10^7^ cells/mL in fetal bovine serum containing 10% dimethyl sulfoxide (DMSO). Frozen cells were thawed and used for the IL-4 ELISPOT assay.

#### 2.7.2. Cytokine ELISPOT

Assays for the cytokines IFN-γ, IL-2, and IL-4 were performed on spleen cells using cytokine kits from Mabtech (Sweden). The kit numbers were 3321M-2H for IFN-γ, 3441-2H for IL-2, and 3311-2A for IL-4. The culture media used was RPMI 1640 supplemented with 10% fetal bovine serum, 50 µM 2-mercaptoethanol, and 1% penicillin–streptomycin. The following antigens obtained from BEI resources were used for the assay: the peptide arrays for the spike protein (S) (cat#NR52402), nucleoprotein (N) (cat#NR52404), membrane protein (M) (cat#NR52403), and envelop (E) protein (cat#NR52405) of the SARS-CoV-2 Wuhan strain. Each individual peptide in the array was dissolved in DMSO, and the arrays were reconstituted into peptide pools with a concentration of 1 mg/mL per peptide. The S protein peptide array contained 181 peptides, so they were made into two stock pools designated as S1 (peptides 1–90) and S2 (peptides 91–181). The remaining three proteins (N, M, and E proteins) were made into one stock pool per protein. Briefly, ELISPOT plates (MAIPSWU10, Millipore) were coated with cytokine-specific monoclonal Abs. The manufacturer-recommended protocols (Mabtech) were used strictly for coating with monoclonal Abs and for developing the ELISPOT plates. After coating the plates and washing, 100 µL/wells of peptide pools were added to the ELISPOT plates. Fresh spleen cells or frozen–thawed spleen cells recovered overnight in culture media (100 µL/well) were immediately added to the wells. The final concentration of peptides in the assay mixtures was 1 µg/mL. The cell concentration for the assays was 2 × 10^5^ cells/well (for IFN-γ and IL-2 assays) or 1 × 10^5^ cells/well (for IL-4 assay). The plates were then incubated at 37 °C in 5% CO_2_ for about 20 h. Mock controls containing everything except for the peptides were prepared and similarly incubated. After a 20-h incubation, the cells were washed and the assay was developed using the manufacturer-recommended protocol (Mabtech). The spots were counted using an automated spot counter (AID ELISPOT Reader, Autoimmun Diagnostika GmbH, Straßberg, Germany). The spots were then normalized based on input cells per well, and the results are expressed as spot-forming units (SFUs) per 10^6^ cells. Data are presented as direct ex vivo cytokine responses and antigen-stimulated responses. ELISPOT data for IFN-γ, IL-2, and IL-4 from the mock control experiments are shown as ex vivo responses, and antigen-stimulated responses represent the data after subtracting the ex vivo responses from the corresponding antigen-stimulated data. 

#### 2.7.3. ELISA Endpoint Determination for Total IgG, IgG1 and IgG2a

The antigens used for measuring total IgG, IgG1, and IgG2a in mouse sera were the recombinant SARS-CoV-2 N protein (Leinco Technologies, Fenton, MO, USA, Cat #: S854), recombinant RBD protein (BEI Resources, Cat #: NR-52946), and recombinant trimeric S protein (Leinco Technologies, Cat #: S848). The secondary antibodies for total mouse IgG, IgG1, and IgG2a were goat anti-mouse IgG (Fc-specific) peroxidase (HRP) (Sigma Aldrich, St. Louis, MO, USA), biotin rat-anti-mouse IgG1, and biotin rat-anti-mouse IgG2a (BD Biosciences, San Jose, CA, USA). Streptavidin-horseradish peroxidase (SA-HRP) (BD Bioscience) was used to detect biotinylated antibodies. 

Flat-bottom ELISA plates were coated with antigens at 1 µg/well (N and RBD) or 0.5 µg/well (trimeric S) in PBS and incubated overnight at 4 °C. Plates were then washed using PBS and blocked using 200 µL/well of 5% skim milk (Difco) for at least 1 h at 37 °C. After washing, serially diluted sera were added to the plates and incubated for 1 h at 37 °C. Plates were washed and the appropriate secondary antibodies to total IgG, IgG1, or IgG2a were added and incubated for 1 h at 37 °C. For measuring total IgG, a goat anti-mouse IgG-HRP conjugate was added and incubated for 1 h at 37 °C. The plates were then washed, and a TMB substrate was added and incubated at room temperature (in dark) for 10 min. After 10 min, stop solution was added, and the plates were read at 450 nm. For measuring IgG1 and IgG2a, the plates were washed and incubated with either the biotinylated anti-mouse IgG1 or anti-mouse IgG2a for 1 h at 37 °C. The plates were then washed with PBS and incubated with streptavidin-horse radish peroxidase for 1 h. The plates were then developed with the TMB substrate for 10 min at room temperature. After adding the stop solution, the plates were read at 450 nm. 

For each experiment, four naive sera were used as negative controls. Mouse anti-SARS-CoV-2 mAb (GenScript, Cat/Clone ID: 10G6H5) and a mouse anti-SARS-CoV-2 N Protein mAb (7B3) (Millipore; Cat: MABX8407) were used as positive controls. Cutoff values were the mean+2.63sd+0.1 of the negative controls for each serum dilution [18]. For test serum, the last serum dilution that scored above the cutoff value was the endpoint titer.

### 2.8. Data Analysis

Data analysis was performed using GraphPad prism 8.3.1. A one-way ANOVA with Tukey’s honestly significant difference (HSD) post hoc test was performed to compare significant differences between groups. Statistical significance is indicated as * *p* < 0.05, ** *p* < 0.01, *** *p* < 0.001, and **** *p* < 0.0001.

## 3. Results

### 3.1. Preparation and Purification of Inactivated SARS-CoV-2 Vaccine

**Figure 1:**
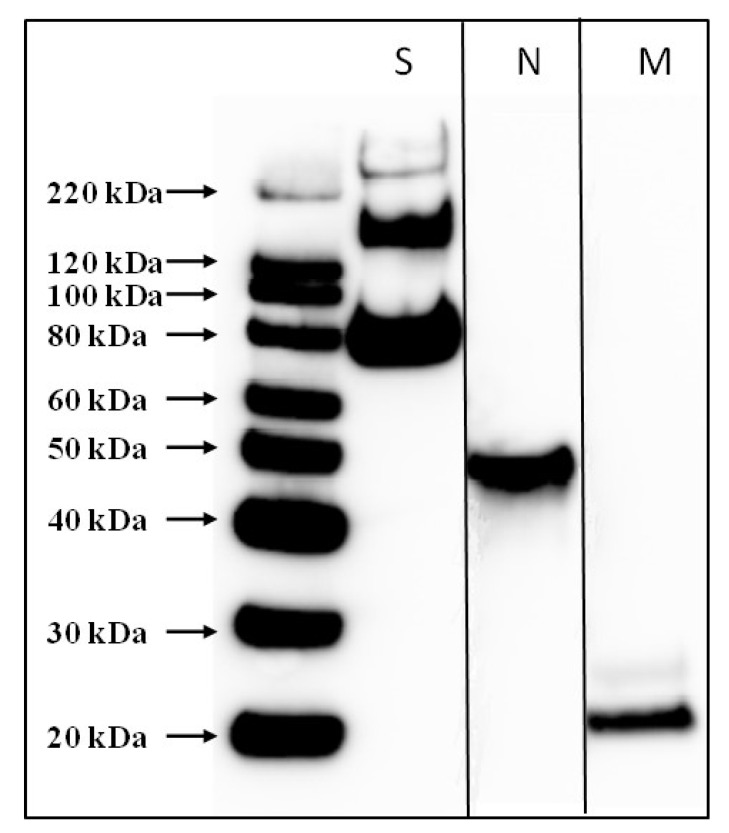
Western blot analysis of purified SARS-CoV-2 PsIV. Presence of SARS-CoV-2 antigens was evaluated by separate Western blots using antibodies specific for spike protein (S), nucleocapsid protein (N), or membrane protein (M). The lanes from the N and M Western blots are shown next to the lanes from the S Western blot. Two distinct bands were observed when spike protein-specific antibodies were used: one for the S protein monomer at 140 Kda and a second band for S1 subunit at 76 Kda.

The SARS-CoV-2 strain nCoV/USA-WA1/2020 was used to prepare a whole-virus psoralen-inactivated vaccine (SARS-CoV-2 PsIV). The vaccine was prepared to a concentration of 10^9^ purified viral particles per mL. The presence of SARS-CoV-2-specific antigens in the SARS-CoV-2 PsIV was confirmed by Western blot analysis using anti-SARS-CoV-2 spike protein-specific, nucleocapsid protein-specific, and envelop protein-specific antibodies (Figure 1).

### 3.2. Antibody Responses to SARS-CoV-2 PsIV in Mice

The immunogenicity of SARS-CoV-2 PsIV was evaluated by immunizing BALB/c mice with different doses of the vaccine in combination with either alum or Advax-2 as an adjuvant. Advax-2 is a delta inulin adjuvant formulated with CpG oligonucleotide that has been shown to improve the immunogenicity and efficacy of a number of vaccines in human clinical trials including against influenza and hepatitis B [19,20,21]. Sera from individual mice in each group at various time points were tested for the presence of SARS-CoV-2-neutralizing antibodies.

**Figure 2:**
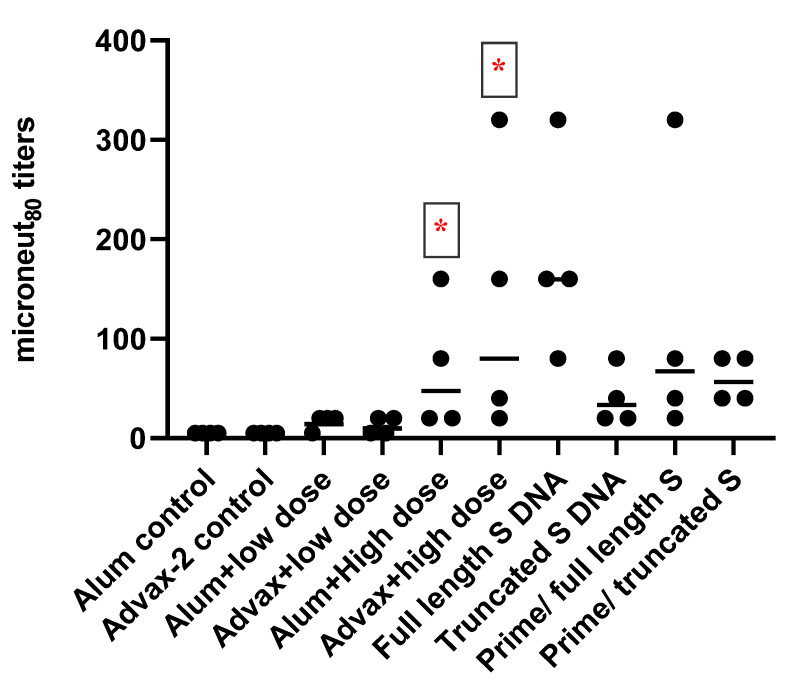
Microneut_80_ data (day 56 sera) from BALB/C mice vaccinated with SARS-CoV-2 PsIV vaccines and DNA vaccines encoding full-length and truncated SARS-CoV-2 spike protein. Circles represent individual mice, and horizontal bars represent the geometric mean for each group. * indicates significant differences (*p* ≤ 0.05) between the adjuvant and PsIV group vs the corresponding adjuvant alone group.

Twenty-eight days after the administration of the second dose of vaccines (day 56 sera), the animals that received the low dose of SARS-CoV-2 PsIVs did not exhibit any significant neutralizing antibody responses (Figure 2). However, animals that received the high dose of SARS-CoV-2 PsIVs elicited robust neutralizing antibody responses, as shown in Figure 2. Animals that received the high dose of SARS-CoV-2 PsIV formulated with the Advax-2 adjuvant exhibited a significantly higher geometric mean neutralizing antibody response than the animals that received the high-dose vaccine with alum. Mice that received the SARS-CoV-2 DNA vaccine encoding the full-length spike protein elicited neutralizing antibody responses, and the geometric mean titer was the highest compared to all other groups. For the prime-boost groups, the geometric mean titers were similar to geometric mean titer in the Advax 2/SARS-CoV-2 PsIV alone group.

**Figure 3:**
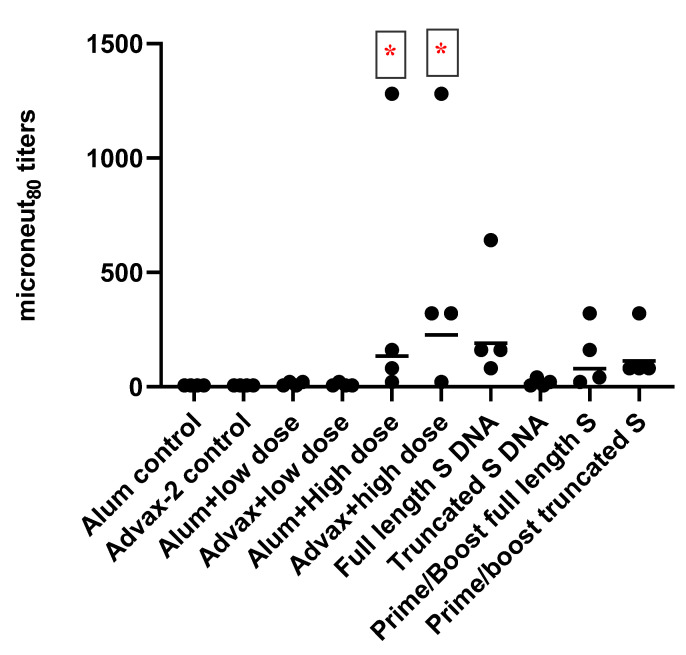
Microneut_80_ data (day 71 sera) from BALB/C mice vaccinated with SARS-CoV-2 PsIV, DNA vaccines encoding the full-length or truncated SARS-CoV-2 spike protein, or both DNA and PsIV vaccines (prime/boost). Circles represent individual mice, and horizontal bars represent the geometric mean for each group. * indicates significant differences (*p* ≤ 0.05) between the adjuvant and PsIV group vs the corresponding adjuvant alone group.

All animals were boosted on day 57 in preparation for measuring the T-cell responses by harvesting spleen cells on day 71 (two weeks after administering the booster dose). For the heterologous prime-boost vaccination groups (9 and 10), the mice that received DNA vaccine on days 1 and 29 received SARS-CoV-2 PsIV on day 57. Mice in the DNA-only groups received a third DNA immunization on day 57. Neutralizing antibody responses on day 71 continued to show statistically superior responses in the high-dose Advax-2 vaccine group compared to the high-dose alum vaccine group (Figure 3). Animals that received the low dose of the SARS-CoV-2 PsIV vaccine did not elicit any neutralizing antibody responses, regardless of the used adjuvant. Mice in the DNA vaccine groups did not exhibit significant increases in neutralizing antibodies after boosting with either the DNA or the PsIV vaccines on day 57. These results indicate that SARS-CoV-2 PsIV could elicit neutralizing antibody responses at higher doses and that the Advax-2 adjuvant better enhances immunogenicity than the alum adjuvant.

#### Total IgG and IgG Subclasses

**Figure 4:**
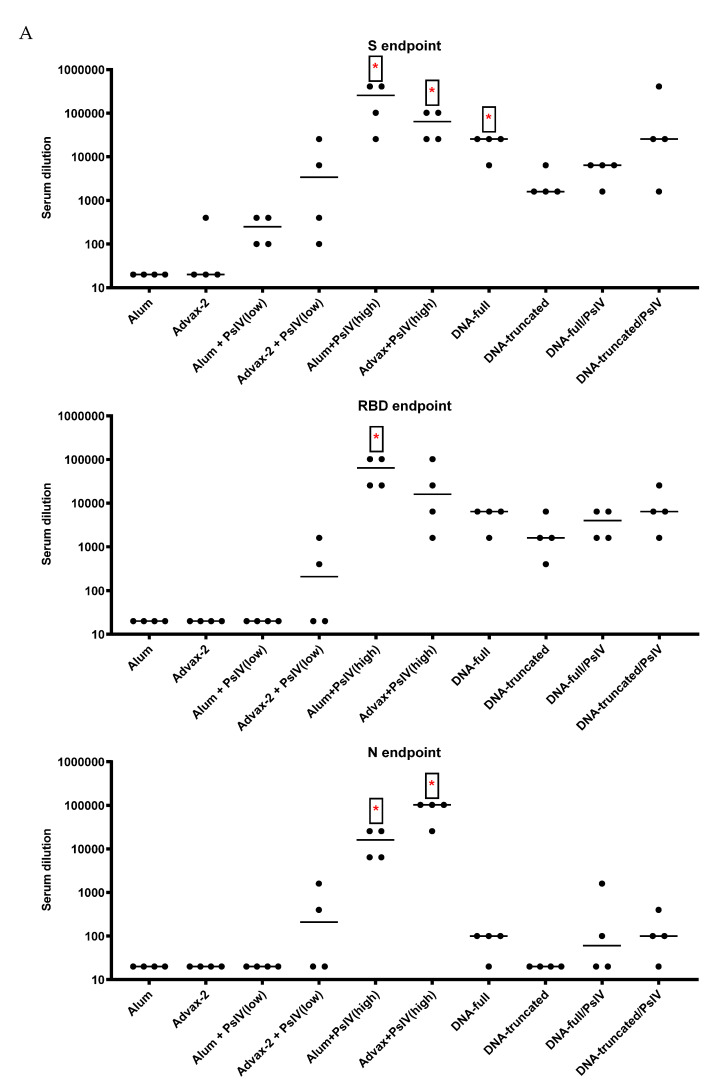

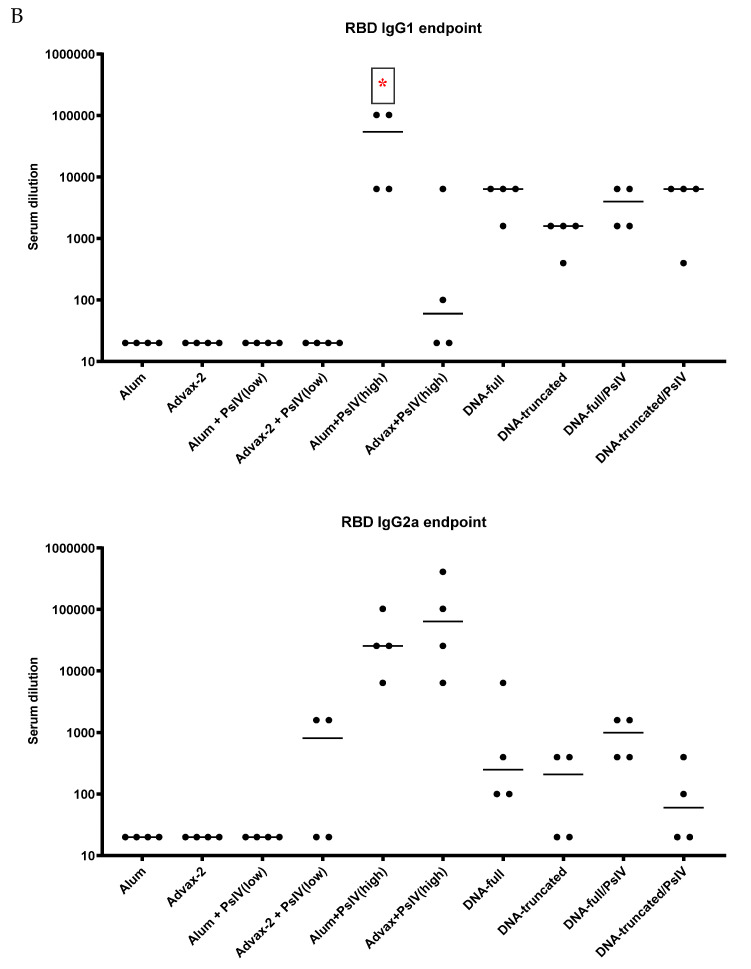
Total IgG, IgG1, and IgG2a endpoint titers to SARS-CoV-2 antigens from day 71 mice sera. (**A**) Total IgG endpoint titers to the spike (S), the receptor-binding domain (RBD), and the nucleocapsid (N) SARS-CoV-2 antigens. (**B**) IgG1 and IgG2a endpoint titers to RBD. Each symbol represents one mouse, and horizontal lines represent group median values. Student’s *t*-tests were used to compare differences between groups. * indicates significant differences between adjuvant and PsIV group vs corresponding adjuvant alone group, as well as between DNA vaccine group vs corresponding DNA/PsIV prime-boost group.

Anti-SARS-CoV-2 IgG antibodies against the spike protein and the receptor-binding domain of the spike protein were detected in all SARS-CoV-2 PsIV- and DNA-vaccinated mice. Mice vaccinated with SARS-CoV-2 PsIV also developed antibodies against the nucleocapsid protein (Figure 4A). A dose-dependent IgG response for SARS-CoV-2 PsIV was evident, as the high dose induced greater IgG titers than the low dose. The DNA vaccine encoding the full-length spike protein induced a higher IgG response than the DNA vaccine encoding the truncated spike protein. The range of the IgG titers varied between the different vaccine groups, with the highest difference being as much as 64-fold. SARS-CoV-2 PsIV in alum elicited the highest IgG1 response, while SARS-CoV-2 PsIV in Advax-2 elicited the highest IgG2a response (Figure 4B), although the difference between the two groups was not significant (*p* = 0.053–0.17). As illustrated in Supplemental Figure S1, the ratio of endpoint titers for IgG2a to IgG1, SARS-CoV-2 PsIV with alum induced a near equivalent RBD IgG2a and IgG1 response, whereas SARS-CoV-2 with Advax-2 induced a predominantly IgG2a response.

### 3.3. T-Cell Responses to SARS-CoV-2 PsIV in Mice

**Figure 5:**
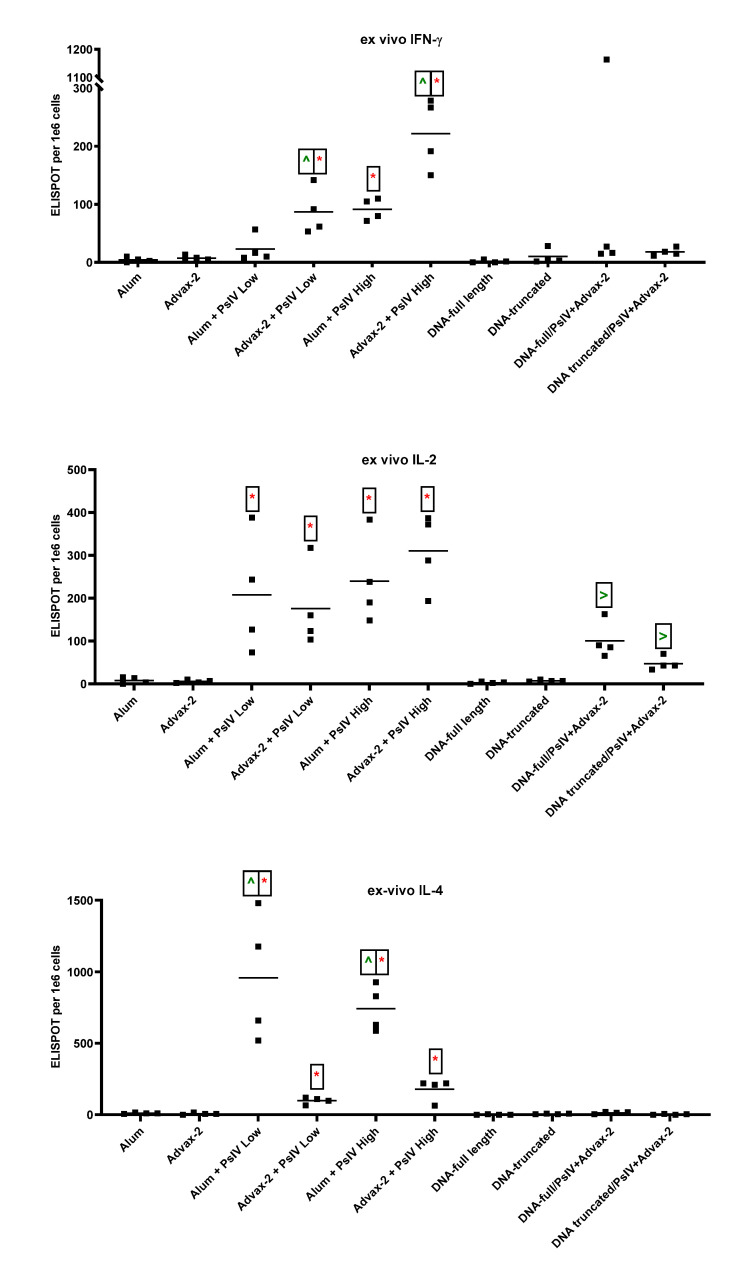
Ex vivo ELISPOT for IFN-γ, IL-2, and IL-4. Mouse splenocytes were used for ELISPOT assays. Data are presented as spot-forming units (SFUs) per 1 × 10^6^ cells. Each symbol represents one mouse, and horizontal lines represent group median values. Student’s *t*-tests were used to compare differences between groups. * indicates significant differences (*p* ≤ 0.05) between the adjuvant and PsIV group vs the corresponding adjuvant alone group. ^ indicates significant differences between alum and PsIV (high or low dose) groups vs the corresponding Advax-2 and PsIV dosage groups. > indicates significant differences between the DNA vaccine alone groups vs the corresponding DNA/PsIV prime-boost groups.

All three cytokines IFN-γ, IL-2, and IL-4 were detected ex vivo without antigen-stimulation in spleen cells isolated from SARS-CoV-2 PsIV-vaccinated mice (Figure 5). The alum and Advax-2 adjuvant alone control groups had minimal IFN-γ (SFUs ≤ 10), IL-2 (SFUs ≤ 15), and IL-4 responses (SFUs ≤ 20). When alum was used as the adjuvant, only the high dose of SARS-CoV-2 PsIV induced a significant IFN-γ response. However, when Advax-2 was used as the adjuvant, both the low and high doses of SARS-CoV-2 PsIV induced significant IFN-γ responses, and a dose-dependent response was observed. In general, SARS-CoV-2 PsIV administration with Advax-2 elicited a significantly higher IFN-γ response than SARS-CoV-2 PsIV administration with alum. Though spleen cells from all animals immunized with SARS-CoV-2 PsIV showed positive IL-4 responses, SARS-CoV-2 PsIV formulated with alum produced significantly higher IL-4 responses than SARS-CoV-2 PsIV formulated with Advax-2. DNA vaccines by themselves or primed with DNA followed by boosting with SARS-CoV-2 PsIV did not elicit any noticeable IFN-γ or IL-4 responses (SFUs ≤ 20). All groups of mice vaccinated with SARS-CoV-2 PsIV showed positive IL-2 responses, whereas mice that received DNA vaccines alone did not. Mice that were primed with DNA vaccines and then boosted with SARS-CoV-2 PsIV also had IL-2 responses. The observed pattern of ex vivo cytokines responses suggests that SARS-CoV-2 PsIV with alum polarized the cytokine response towards a Th2-type response, while SARS-CoV-2 PsIV with Advax-2 polarized the cytokine response towards a Th1-type response.

**Figure 6:**
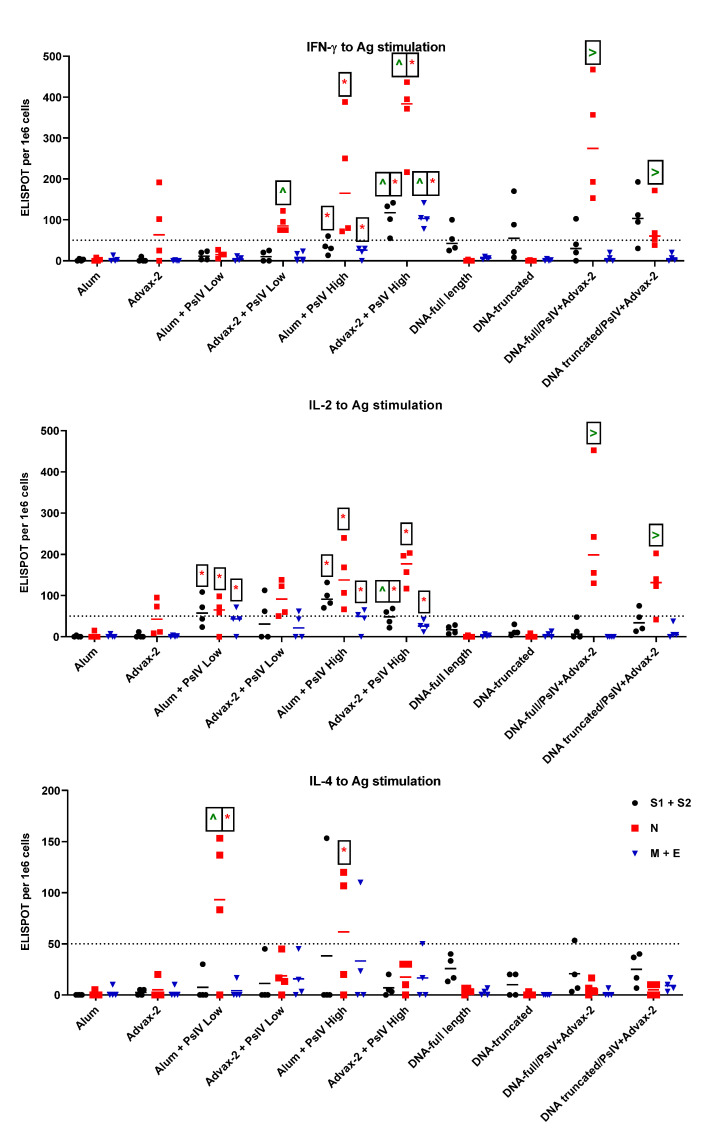
IFN-γ, IL-2, and IL-4 response to antigen stimulation. Mouse splenocytes were stimulated with peptide pools representing whole-length spike (divided into two pools S1 and S2), nucleocapsid (N), membrane (M), and envelope (E) proteins. Antigen-specific responses to the S peptide pool, the N peptide pool, and the M and E peptide pools are shown in black, red, and blue, respectively. Data are presented as SFUs per 1 × 10^6^ cells. Each symbol represents one mouse, and horizontal lines represent group median values. Student’s *t*-tests were used to compare differences between groups for the corresponding antigens. * indicates significantly differences (*p* ≤ 0.05) between the adjuvant and PsIV groups vs the corresponding adjuvant alone group. ^ indicates significant differences between alum and PsIV high- or low-dose groups vs the corresponding Advax and PsIV dosage groups. > indicates significant differences between the DNA vaccine alone groups vs the corresponding DNA/PsIV prime-boost groups.

In addition to ex vivo cytokine-producing T effector cells, memory T cells can also respond to antigen stimulation to produce cytokines. In order to assess memory cells, three SARS-CoV-2 antigen pools, S1+S2, N, and M+E were used to stimulate cells for ELISPOT responses. For these analyses, a SFU value of 50 or more was considered a good response. Alum and Advax-2 alone showed minimal IL-4 (SFUs ≤ 20), IL-2 (SFUs ≤ 15), and IFN-γ (SFUs ≤ 25) responses to all three antigens, with the exception of two mice in the Advax-2 adjuvant group (Figure 6). These two mice showed positive IL-2 and IFN-γ responses, with SFUs > 50 to the N peptide pool. For IFN-γ, a low dose of SARS-CoV-2 PsIV in alum induced a low response, with overall SFUs ≤ 27, whereas a high dose of SARS-CoV-2 PsIV in alum showed a good response to N peptide pools (SFUs = 75–122), though not to other antigens. A low dose of SARS-CoV-2 PsIV in Advax-2 elicited a good response to N antigens (SFUs = 72–388), while the high dose of SARS-CoV-2 PsIV in Advax-2 elicited good responses to all three antigens. SARS-CoV-2 PsIV in Advax-2 elicited significantly higher IFN-γ responses than those of SARS-CoV-2 PsIV in alum. DNA vaccines induced IFN-γ responses to the S peptide pool, but after boosting with SARS-CoV-2 PsIV, a response to the N peptide pool was also observed. In contrast to IFN-γ responses, only SARS-CoV-2 PsIV in alum elicited IL-4 responses. All the other vaccine groups showed responses that were mostly below 50 SFUs. All SARS-CoV-2 PsIV vaccine groups showed IL-2 responses to antigen stimulation. The DNA vaccines alone did not elicit IL-2 responses, but after boosting with SARS-CoV-2 PsIV, the prime-boost groups showed IL-2 responses, primarily to the N-peptide pool. Overall, the pattern of cytokine ELISPOT responses to antigen-stimulation appeared to be similar to that of the ex vivo cytokine responses, suggesting that SAR-CoV-2 PsIV with alum promoted a Th2-type response while SARS-CoV-2 PsIV with Advax-2 promoted a Th1-type response.

## 4. Discussion

We prepared and evaluated a purified psoralen-inactivated whole-virus SARS-CoV-2 vaccine in BALB/c mice. Currently, this vaccine is one of the few inactivated whole-virus vaccines developed to target the whole SARS-CoV-2 virus, in contrast to the hundreds of vaccines targeting just the spike protein of the virus. The other SARS-CoV-2-inactivated whole-virus vaccines are prepared using β-propiolactone, a commonly used chemical to inactivate viruses [7,8]. This compound inactivates viruses by altering both nucleic acid and surface proteins, similar to formalin. Unlike β-propiolactone and formalin, psoralen inactivates viral pathogens at the nucleic acid level, thereby leaving surface viral proteins mostly undisturbed [11,12]. Using dengue virus, psoralen-inactivated vaccines generated superior neutralizing antibody responses compared to formalin-inactivated vaccines [12]. Employing a platform based on psoralen inactivation to develop a SARS-CoV-2 vaccine was therefore a logical approach.

SARS-CoV-2 PsIV was evaluated with both the traditional alum adjuvant and the novel adjuvant Advax-2. The results of our studies in mice indicate that SARS-CoV-2 PsIV is capable of eliciting robust neutralizing antibody responses. The immunogenicity of SARS-CoV-2 PsIV was significantly better when formulated with the Advax-2 adjuvant than with the alum adjuvant (Figure 2 and Figure 3). These results are consistent with previously reported data for other vaccines including influenza and West Nile virus vaccines [21,22]. In earlier preclinical studies of other SARS coronavirus vaccines, the use of alum as an adjuvant contributed to respiratory pathology by inducing Th2 immune responses within the respiratory tract, whereas Advax-2 primarily elicited Th1 responses, resulting in the prevention of vaccine-associated lung immunopathology [23]. The immunogenicity results observed in this study for SARS-CoV-2 PsIV with alum and Advax-2 in mice similarly showed that Advax-2 promoted a Th1 response while alum promoted a Th2 response (Figure 4B, Figure 5 and Figure 6). This suggests that a combination of SARS-CoV-2 PsIV and Advax-2 would be an ideal vaccine–adjuvant combination with minimal risk for vaccine-related lung immunopathology upon re-exposure to the virus. 

The neutralizing antibody responses elicited by SARS-CoV-2 PsIV observed in our preclinical study are at least similar to, if not better than, the responses observed for other whole-virus-inactivated SARS-CoV-2 vaccines that are currently in clinical trials, especially considering that the highest dose of SARS-CoV-2 PsIV tested in this study was 10^7^ particles (equivalent to low ng SARS-CoV-2 proteins/dose). β-propiolactone-inactivated SARS-CoV-2 vaccines (BBIBP-CorV or PiCoVacc) have been evaluated at significantly higher doses (2–8 µg of proteins/dose) in animal models [7,8,24]. When 10^5^ particles of SARS-CoV-2 PsIV were used to immunize the animals, little neutralizing antibody responses were seen. However, the administration of 10^7^ particles of SARS-CoV-2 PsIV resulted in a robust neutralizing antibody response. Therefore, dose-dependent neutralizing antibody responses are observed in mice. Currently, we are in scale-up process development to produce larger quantities of the SARS-CoV-2 PsIV vaccine at higher concentrations (10^11^–10^12^ particles/mL) to conduct a dose-escalation and efficacy study in nonhuman primates.

While the SARS-CoV-2 PsIV vaccine generated superior neutralizing antibody responses in mice when compared to the DNA vaccines and the prime-boost approach in our study, it is possible that the 50 µg/dose of DNA or the intradermal administration of DNA in mice may not be the optimal dose or route of administration of DNA vaccines in mice. However, we believe that the 50 µg/dose of DNA vaccines is generally the optimal dose in mice, as has been observed with dengue DNA vaccines in our lab (unpublished data). It is possible other routes of DNA vaccine administration such as IM and electroporation may give better neutralizing antibody responses and therefore need to be evaluated further [25]. 

In summary, we demonstrated that a psoralen-inactivated SARS-CoV-2 vaccine generates robust neutralizing antibody and T-cell responses to spike and nuclear proteins in mice. It currently is unclear whether immune responses for other viral proteins, besides the spike protein, are required for long-term protection against SARS-CoV-2 infection. It is possible that natural immune responses to the entire virus (live or inactivated) could elicit some non-protective Ab responses to immunodominant but not protective epitopes. However, detection of neutralizing antibody responses against SARS-CoV-2 S and N proteins in most COVID-19 patients within three weeks of infection has recently been reported [6]. Detection of memory CD4^+^ and CD8^+^ T cells primed against SARS-CoV-2 S, N, and M proteins of recovered COVID-19 patients has also been reported [26]. Therefore, as more information regarding correlation of protection and requirements for long-term immunity against SARS-CoV-2 is uncovered, our psoralen-inactivated whole-virus vaccine may provide advantages over other vaccines that only target the SARS-CoV-2 spike protein. Furthermore, formulating SARS-CoV-2 PsIV with the Advax-2 adjuvant promotes a strong Th1 response, which could be beneficial to avoid the excess Th2 bias that has been reported to be associated with vaccine-related lung immunopathology in previous small animal preclinical studies of other inactivated whole-virus coronavirus vaccines. 

## Figures and Tables

**Table 1:** Vaccine groups and dosage for immunogenicity study in mice. Control animals received either alum or Advax-2 in PBS (Groups 1 and 2, respectively). Two doses of the vaccines (with either 0.2% Alhydrogel or 1 mg of Advax-2 per dose) were intradermally administered to all animals (first dose on day 1 and second dose on day 29). Animals in the prime/boost group also received a dose of SARS-CoV-2 PsIV on day 57. DNA vaccines (without any carrier) were intradermally administered.

Groups	Adjuvant and Vaccine	No. of Animals	Dose
1	Alum and PBS	4	N/A
2	Advax-2 and PBS	4	N/A
3	Alum and SARS-CoV-2 PsIV (low dose)	4	10^5^ particles of SARS-Cov-2 PsIV
4	Advax-2 and SARS-CoV-2 PsIV (low dose)	4	10^5^ particles of SARS-Cov-2 PsIV
5	Alum and SARS-CoV-2 PsIV (high dose)	4	10^7^ particles of SARS-Cov-2 PsIV
6	Advax-2 and SARS-CoV-2 PsIV (high dose)	4	10^7^ particles of SARS-Cov-2 PsIV
7	DNA vaccine encoding full-length SARS-CoV-2 spike protein	4	50 µg of DNA
8	DNA vaccine encoding truncated SARS-CoV-2 spike protein	4	50 µg of DNA
9	Prime/Boost (2 doses of DNA; full-length S protein and PsIV)	4	50 µg of DNA (2 doses) and 10^7^ particles of SARS-Cov-2 PsIV
10	Prime/Boost (2 doses of DNA; truncated S protein and PsIV)	4	50 µg of DNA (2 doses) 10^7^ particles of SARS-Cov-2 PsIV

## Supplementary Materials

The following are available online at https://www.mdpi.com/article/10.3390/pathogens10050626/s1, Figure S1: Ratio of IgG2a to IgG1 endpoint titers to RBD. Each symbol represents one mouse and the horizontal line represents geometric mean value.

## Disclaimer

The views expressed in this article reflect the results of research conducted by the authors and do not necessarily reflect the official policy or position of the Department of the Navy, the Department of Defense, or the United States Government. The authors are military service members or federal employees of the United States Government. This work was prepared as part of their official duties. Title 17 U.S.C. 105 provides that ‘Copyright protection under this title is not available for any work of the United States Government.’ Title 17 U.S.C. 101 defines a U.S. Government work as work prepared by military service members or employees of the U.S. Government as part of those persons’ official duties.
