# Supplementary material for: Immunogenicity of Adjuvanted Psoralen-Inactivated SARS-CoV-2 Vaccines and SARS-CoV-2 Spike Protein DNA Vaccines in BALB/c Mice
Source: Pathogens. 2021 May 19;10(5):626. doi: 10.3390/pathogens10050626 (PMC8160882; doi:10.3390/pathogens10050626)
Supplement: Supplementary file 1 [file pathogens-10-00626-s001.zip › pathogens-1192515-supplementary.pdf]

Supplemental Figure 1.

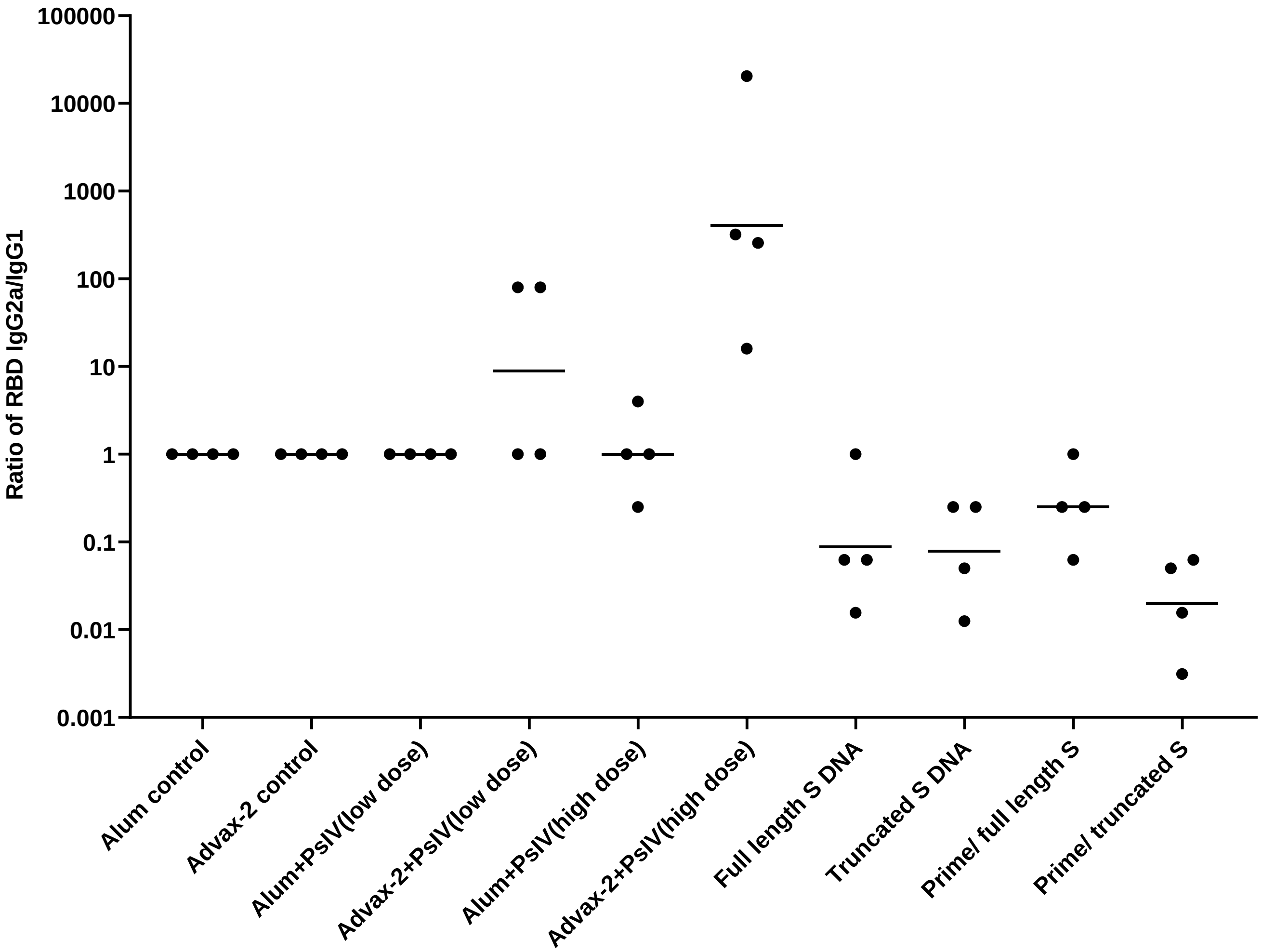

Figure Legend S1. Ratio of IgG2a to IgG1 endpoint titers to RBD. Each symbol represents one mouse and the horizontal line represents geometric mean value.
